# Uptake and Intracellular Trafficking of Superantigens in Dendritic Cells

**DOI:** 10.1371/journal.pone.0066244

**Published:** 2013-06-14

**Authors:** María B. Ganem, Mauricio C. De Marzi, María J. Fernández-Lynch, Carolina Jancic, Mónica Vermeulen, Jorge Geffner, Roy A. Mariuzza, Marisa M. Fernández, Emilio L. Malchiodi

**Affiliations:** 1 Cátedra de Inmunología and Instituto de Estudios de la Inmunidad Humoral (IDEHU), CONICET-UBA, Facultad de Farmacia y Bioquímica, Universidad de Buenos Aires, Buenos Aires, Argentina; 2 Departamento de Ciencias Básicas, Universidad Nacional de Luján, Luján, Buenos Aires, Argentina; 3 Departamento de Inmunología, Instituto de Investigaciones Hematológicas, Academia Nacional de Medicina, Buenos Aires, Argentina; 4 University of Maryland Institute for Bioscience and Biotechnology Research, W. M. Keck Laboratory for Structural Biology, Rockville, Maryland, United States of America; Karolinska Institutet, Sweden

## Abstract

Bacterial superantigens (SAgs) are exotoxins produced mainly by *Staphylococcus aureus* and *Streptococcus pyogenes* that can cause toxic shock syndrome (TSS). According to current paradigm, SAgs interact directly and simultaneously with T cell receptor (TCR) on the T cell and MHC class II (MHC-II) on the antigen-presenting cell (APC), thereby circumventing intracellular processing to trigger T cell activation. Dendritic cells (DCs) are professional APCs that coat nearly all body surfaces and are the most probable candidate to interact with SAgs. We demonstrate that SAgs are taken up by mouse DCs without triggering DC maturation. SAgs were found in intracellular acidic compartment of DCs as biologically active molecules. Moreover, SAgs co-localized with EEA1, RAB-7 and LAMP-2, at different times, and were then recycled to the cell membrane. DCs loaded with SAgs are capable of triggering *in vitro* lymphocyte proliferation and, injected into mice, stimulate T cells bearing the proper TCR in draining lymph nodes. Transportation and trafficking of SAgs in DCs might increase the local concentration of these exotoxins where they will produce the highest effect by promoting their encounter with both MHC-II and TCR in lymph nodes, and may explain how just a few SAg molecules can induce the severe pathology associated with TSS.

## Introduction

Bacterial superantigens (SAgs) comprise a large family of exotoxins produced mainly by *Staphylococcus aureus* and *Streptococcus pyogenes* [1]. SAgs produced by *S. aureus* include SEA through SER, excluding F, and TSST-1, and constitute the major cause of food poisoning in humans. These SAgs induce diarrhea, emesis, and systemic intoxication, leading to a severe condition known as toxic shock syndrome (TSS). This syndrome includes high fever of sudden outbreak, rash, diarrhea and, in some cases, renal and lung failure that may end in death. *S. pyogenes* SAgs including SPEA and SSA, cause scarlet fever, pyrogenicity, and a fulminant illness known as streptococcal TSS, which displays features similar to staphylococcus TSS. SAgs are classified as Category B priority agents by the Centers for Disease Control and Prevention because of their potential use in bioterrorism and biological warfare.

The biological effects of SAgs rely on their capacity to induce the massive release of inflammatory cytokines such as IL-2, INF-γ, and TNF-α. This cytokine storm has been shown to depend on the interaction of the SAg with the T cell receptor (TCR) present on T cells, and major histocompatibility complex class II molecules (MHC-II) on antigen-presenting cells (APCs) [Reviewed in 2]. In conventional antigen processing, antigens are incorporated by APCs, processed into peptide fragments, and the fragments presented to T cells bound to MHC-II molecules. T cells will only respond if they recognize the MHC-II molecule and the specific antigenic peptide being presented. By contrast, SAgs stimulate T cells, as unprocessed intact proteins, by simultaneously binding MHC-II and TCR.

The direct interaction of SAgs with TCR and MHC-II has been demonstrated by several biophysical methods, including surface plasmon resonance (SPR), calorimetry, and analytical ultracentrifugation [3]–[14], and by X-ray crystallography of binary [15]–[22] and ternary [23] complexes. These structures revealed how SAgs circumvent the normal mechanism for T cell activation by peptide/MHC and how they stimulate T cells expressing TCR β chains from a number of different families, resulting in polyclonal T cell activation. Recently, the crystal structure of the ternary complex between the staphylococcal superantigen SEH and its human receptors MHC-II and TCR was obtained. Unlike the conventional SAg engagement of the TCR Vβ domain, SEH predominantly interacts with the Vα domain [24]. Collectively, these studies support the current paradigm of direct and simultaneous interaction of SAgs with TCR on the T cell and with MHC-II on the APC, thereby circumventing intracellular processing to trigger T cell activation. However, an unexplored issue is how SAgs reaches the site where APCs and T cells are in close contact, namely lymphoid tissues, and where simultaneous binding of TCR and MHC-II actually occurs.

Dendritic cells (DC) are professional APC located at mucosal and epithelial surfaces where they perform a sentinel function looking for foreign antigens. DCs actively take up antigens, process them, and, following activation, initiate maturation and migration to the lymph nodes where they activate T lymphocytes to trigger the adaptive immune response [25]. DCs coat nearly 95% of mucosal and skin surfaces, where many streptococcal and staphylococcal infections occur, and, thus, they are the most probable candidate to interact with SAgs. However, immature DCs continuously take up antigens from their microenvironment, which implies continuous cell membrane internalization. This activity could represent a limitation to superantigenicity, since SAgs could be internalized, as many other antigens, and not remain on the cell surface to interact with TCR. In addition, it is not clear what would happen to SAgs interacting with an immature APC if they were incorporated, since such antigens are normally processed into peptides, which would abrogate SAg activity.

Several studies have analyzed the interaction of bacterial SAgs with DCs. Incorporation by DCs of the streptococcal SAg SMEZ-2, conjugated or not to OVA, was demonstrated by confocal microscopy [26]. Human monocyte-derived DCs showed increased expression of maturation markers after 48 h of incubation with SEB [27]. Another study used a cocktail of SAgs (TSST-1, SEE, SEA, SEC and SED) to induce migration of immature DCs, which required CD4^+^ T cells [28]. Other studies have shown that DC maturation by SAgs depends on the presence of T cells [29]–[33]. Most of these studies have assumed that SAgs interact with MHC-II on the cell membrane of DCs in the extracellular space. However, they have not addressed whether SAgs are incorporated by DCs in the same manner as conventional antigens, and, if so, how SAgs escape antigen processing.

Here, we analyzed SAg interactions with mouse DCs and demonstrated that: 1- DCs take up SAgs; 2- SAgs alone are not able to trigger DC maturation; 3- SAg can be found in the intracellular membrane system of DCs as biologically active molecules; 4- SAgs are present in the acidic compartment and remain active; 5- SAgs co-localize with EEA1, RAB-7 and LAMP-2 at different times after incorporation; 6- SAgs are recycled to the cell membrane of DCs as soon as 2 h after uptake; 7- DCs loaded with SAgs are capable of triggering potent *in vitro* lymphocyte proliferation; and 8- DCs pulsed with SAgs and injected into mice stimulate T cells bearing the proper TCR β chain in draining lymph nodes. Collectively, these results suggest that intracellular trafficking of SAgs in DCs serves to increase the local concentration of SAgs and promote their encounter with both MHC-II on APCs and TCR on T cells in lymph nodes. In addition, the interaction of SAgs with DCs may explain how just a few SAg molecules secreted by infecting bacteria can induce the severe pathological effects associated with TSS and other SAg-mediated disorders.

## Results

### SAgs are Efficiently Incorporated by DCs without Inducing DC Maturation

**Figure 1:**
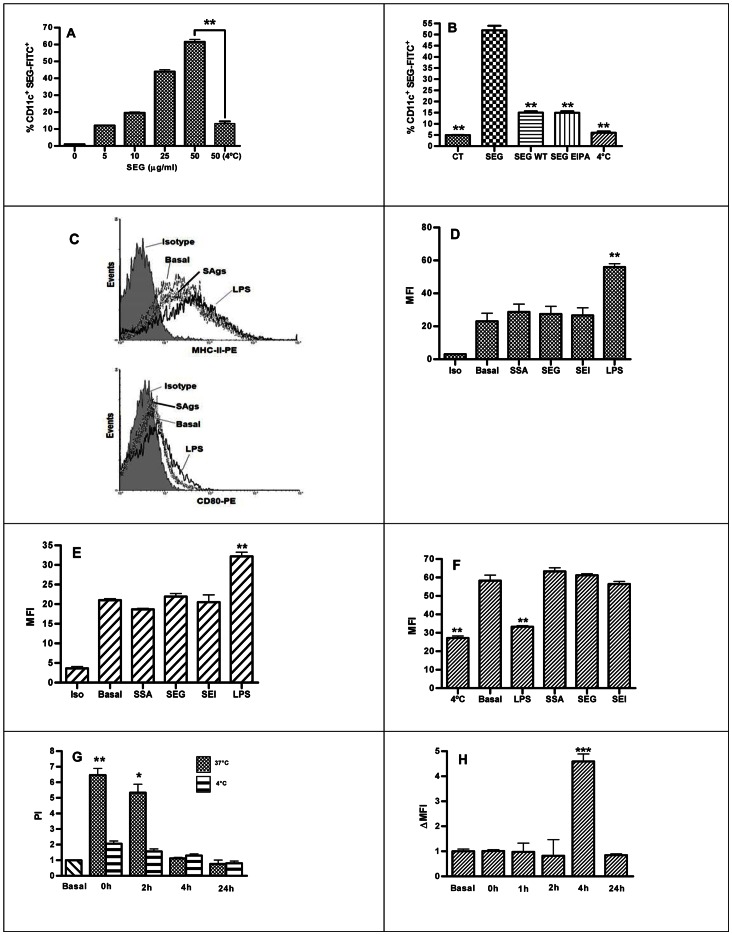
SAg incorporation in DCs and cell activation markers. Bone marrow DCs were pulsed 1 h with SEG-FITC, washed, and immunolabeled with anti CD11c-PE. (**A**) Percentage of DCs incorporating different concentration of SEG-FITC at 37°C and 4°C *vs.* non-pulsed DCs as control. (**B**) Percentage of DCs showing inhibition of SEG-FITC internalization by wortmannin (WT) and 5-ethyl-N-isopropyl amiloride (EIPA) added at final concentrations of 100 nM and 50 µM, respectively, 15 min before SAg-FITC at 50 µg/ml. (**C**) Representative histograms of the phenotype of DCs in the presence of SAgs. (**D**) Cell surface MHC-II molecules on DCs treated with SSA, SEG, SEI or LPS for 24 h, compared to non-treated basal control, and expressed in MFI. (**E**) CD80 on SAgs- or LPS-treated DCs compared to non-treated. (**F**) Endocytosis of OVA-FITC by SAg or LPS pre-treated DCs. (**G**) DCs were incubated for 1 h with SEG at 37°C or 4°C as control, washed, and cultured for additional periods from 0 to 24 h. At each endpoint, cells were washed and disrupted in order to isolate the endosomal/lysosomal compartment. These fractions were incubated with mouse splenocytes to assess proliferative activity by ^3^H-Thy incorporation in triplicate. The proliferation index (PI) was calculated as cpm/basal cpm. Basal represent the proliferation of T cells stimulates by DCs incubated in the absence of SAg. (**H**) SEG-pulsed DCs cultured for additional periods of 0 to 24 h. At each endpoint, cells were washed and immunolabeled with mouse anti-SEG polyclonal antibodies and anti-mouse-FITC, as second antibody to evaluate the presence of SEG on the cell surface. Figures show a representative experiment of 3–5. *p<0.05, **p<0.01.

In order to determine whether mouse DCs are able to internalize SAgs, cells were incubated with FITC-labeled *S. aureus* SEG and SEI, and *S. pyogenes* SSA, and immunolabeled with CD11c-PE. Cells were analyzed by FACs in the presence of trypan blue, and compared to non-pulsed DCs as control. **Fig. 1A** shows that a high percentage of DCs internalized SEG-FITC after a pulse in a dose-dependent manner, and that this process was markedly reduced when the pulse was carried out at 4°C. Unlabeled SEG was not able to inhibit the uptake of SEG-FITC (result not shown), suggesting that a specific saturable receptor was not involved. The phosphoinositide 3-kinase (PI3K) inhibitor wortmannin (WT) and the ion exchange inhibitor 5-[N-ethyl-N-isopropyl] amiloride (EIPA) were used to elucidate whether macropinocytosis was responsible for SAg incorporation. **Fig. 1B** shows a strong reduction of SEG-FITC internalization when the pulse was performed in the presence of WT (71.0% of inhibition) or EIPA (71.2%). The dot plot analysis of CD11c^+^ versus FITC^+^ cells for a concentration of SEG-FICT 50 µg/ml in the different conditions is shown in **Fig. S1** in **File S1**. Similar results were obtained with SEI and SSA (data not shown). In addition, we cultured DCs for longer time in the cold or in the presence of WT, DMSO or EIPA with not changes in viability (results not shown).

Next, we analyzed whether SAgs are able to induce phenotypic maturation of murine DCs. LPS and three SAgs (SEG, SEI and SSA) were incubated with DCs and expression of CD80, CD86 and MHC-II was analyzed by cytometry after 24 h of incubation. In contrast to LPS, SAgs did not induce any change in the expression of MHC-II (**Fig. 1C** upper panel and **Fig. 1D**), CD80 (**Fig. 1C** lower panel and **Fig. 1E**), CD86 (**Fig. S2** I in **File S1**) and CD40 (**Fig. S2** J in **File S1**). Percentage of positive cells is shown in **Fig. S2** **D**, **E** and **F** in **File S1**. However, when DCs underwent maturation after treatment with LPS, their ability to incorporate SAgs was lost (data not shown). Consistent with these results, we found that endocytosis of OVA-FITC was inhibited in LPS-treated DCs, as expected, but not impaired in SAg-treated DCs (**Fig. 1F**). Together, these results indicate that macropinocytosis plays a major role in SAgs uptake by DCs and that SAgs do not induce maturation of DCs. Since we did not find any difference among the three SAgs analyzed, we conducted all subsequent experiments with SEG, unless noted otherwise.

To evaluate the presence of SAgs in the intracellular compartment, and to investigate whether these exotoxins resist degradation, DCs were pulsed for 1 h with SEG as previously described, washed, and cultured from 0 to 24 h. After incubation, the endosome/lysosome fraction was isolated as described in Materials and Methods. The endosome/lysosome fraction was isolated and lysed by repeated cycles of freezing and thawing, and exposed to syngeneic splenocytes to assess cell proliferation as a biological method for detecting SAgs. SEG was effectively detected in extracts carried out immediately after the pulse and also 2 h later. However, extracts isolated at 4 and 24 h after pulsing showed no signs of biologically active SEG (**Fig. 1G**). The absence of SAg activity at 4 and 24 h could be attributed to both degradation of the SAg, migration to other subcellular locations such as the cytoplasm or cell membrane, or release into the medium. To test for the presence of SAg on the cell membrane, we carried out indirect immunolabeling of SEG with mouse anti-SEG polyclonal antibodies at different time points. SEG could not be detected on the cell membrane at 0 and 2 h after the pulse, which is in agreement with the presence of the SAg in the endosomal/lysosomal compartment. Surprisingly, SEG was detected on the membrane 4 h after the pulse, coincident with the loss of detection of SEG in the intracellular compartment (**Fig. 1H**). After 24 h, no active SAg could be detected in the endosomal/lysosomal compartment or on the cell membrane. These experiments show that SAgs conserve their molecular architecture sufficiently to be recognized by antibodies and stimulate syngeneic splenocytes during their presence in endosomal/lysosomal compartments and further exposure on the membrane.

### Incorporated SAgs Traffic into Acidic Organelles

**Figure 2:**
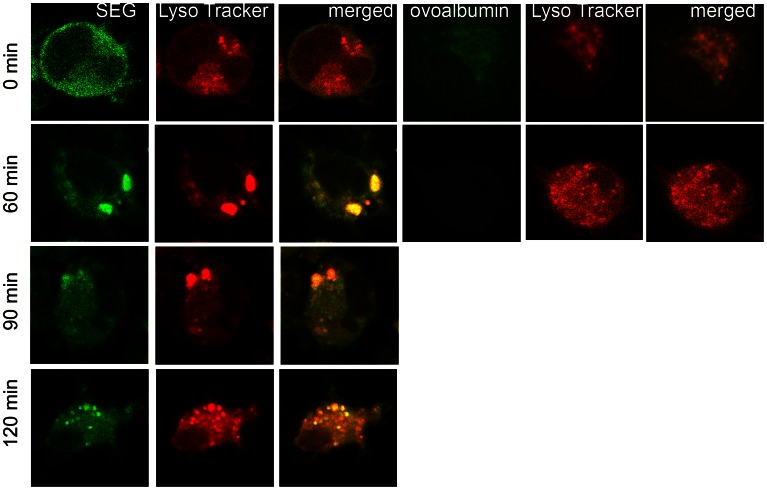
After incorporation in DCs, SEG co-localize with LysoTracker in acidic organelles. DCs were cultured on poly-L-lysine treated slides, pulsed 1 h at 37°C with 50 µg/ml SEG-FITC and 50 µM Lysotracker RED (Molecular Probes, Invitrogen), washed and cultured further for different time periods (0–120 min). Fluorescently labeled cells were fixed with paraformaldehyde at 4°C, and cover slides mounted using anti-fade mounting media (Vector Laboratories). Image capture was performed using a Nikon C1 confocal laser scanning microscope with a PlanApo 60× Oil AN1.40 lens. For detection of FITC a 490 nm laser was used. A 546-nm excitation wavelength and a 590-nm emission wavelength were used for LysoTracker. Analysis by confocal microscope (6000×) shows that SEG barely co-localized at acidic compartment at time point 0, but clearly co-localized with LysoTracker at the 60 min time point and later. Cells pulsed with 50 µg/ml OVA-FITC for 1 h showed co-localization of OVA with Lysotracker at time point 0, but 60 min later only LysoTracker remained inside the cell. Figure shows a representative experiment of 3.

Since endosomal vesicles usually fuse with lysosomes that contain enzymes at acid pH, laser scanning confocal microscopy was used to investigate whether SAgs taken up by DCs were located in acidic vesicles. DCs were pulsed for 1 h at 37°C with SEG-FITC and LysoTracker Red, washed four times, and cultured from 0 to 120 min. Cells were collected at different time points, washed, and fixed. Confocal images were captured using a PlanApo 60× Oil AN 1.40 immersion objective. Controls with non-pulsed cells (autofluorescence) and cells pulsed at 4°C were performed in parallel (data not shown). **Fig. 2** shows that, after a 1 h pulse (time point 0), SEG was incorporated but hardly any SAg co-localized with LysoTracker Red, which was inside acidic organelles in the cytoplasm. However, 40 (not shown) to 60 min after pulse, SEG co-localized preferentially with the LysoTracker probe in a few large acidic organelles. The bright spots represent increased LysoTracker staining and indicate regions of high lysosomal activity. The label faded with time, but SEG could still be observed in smaller acidic organelles distributed throughout the cytoplasm at 120 min after pulse. By contrast, controls conducted under similar conditions and time points with OVA and LysoTracker showed that both co-localized in large organelles after a 1 h pulse. However, 60 min after the 1 h pulse little or no OVA could be detected, whereas LysoTracker was still visible inside vesicles (**Fig. 2**).

### SEG Co-localizes with EEA-1, LAMP-2 and RAB-7 in the Lysosomal Compartment

**Figure 3:**
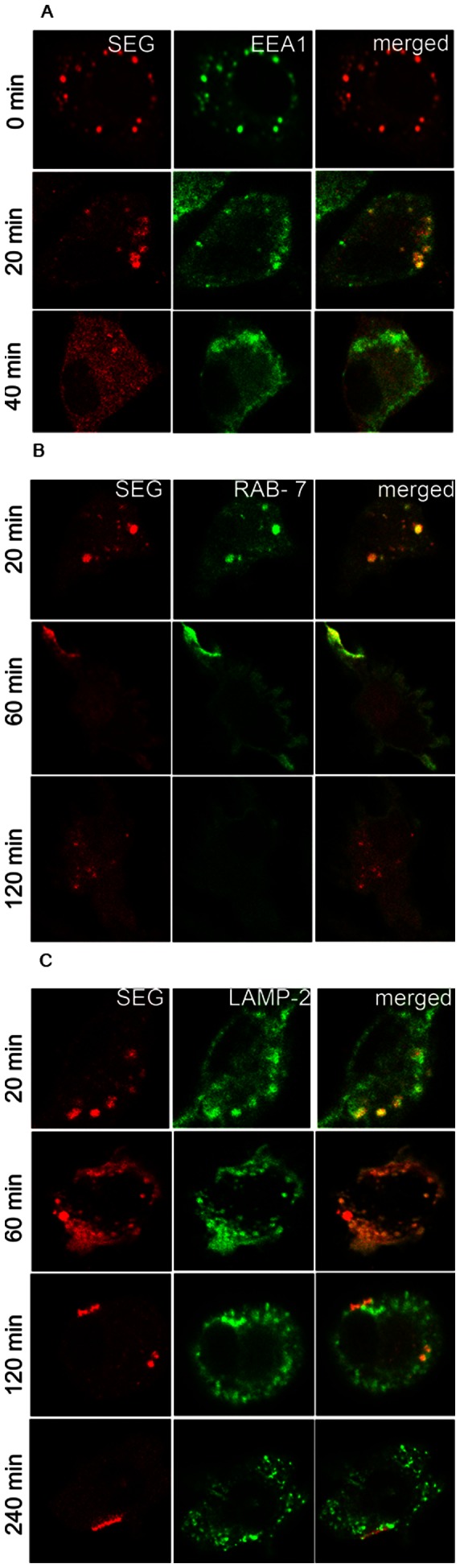
After incorporation in DCs, SEG is located in vesicles inside DCs and then is exposed on cell membrane. DCs were cultured on poly-L-lysine treated slides, pulsed 1 h at 37°C with SEG, washed, and cultured further for different time periods (0–240). Indirect Immunolabeling of intracellular SEG and the organelle-specific markers was conducted with mouse polyclonal serum to SEG concurrently with polyclonal rabbit anti-EEA1, anti-RAB-7 or anti-LAMP-2, (Sigma Aldrich, St. Louis, MO). Cells were then incubated with FITC-labeled anti-rabbit and rodamine A-labeled anti-mouse for analysis by confocal microscopy. Image capture was performed using a Nikon C1 confocal laser scanning microscope with a PlanApo 60× Oil AN1.40 lens. For detection of rhodamine, a 543 nm laser and 490 nm laser for FITC were used. Controls of non-pulsed cells were performed in parallel. (**A**) Immediately (0 min) and 20 min after the 1 h pulse, the location of SEG (red) was exclusively intracellular and showed strong co-localization with EEA1 (green), which was lost at 40 min of culture. At this latter time, SEG was still inside vesicles, but EEA1 was scattered throughout the cell cytoplasm. (**B**) Co-localization of SEG with RAB-7 starts at 20 min after 1 h pulse with SAg. At 60 min, a portion of SEG could be seen in RAB-7^+^ small vesicles scattered throughout the cytoplasm and also concentrated in clusters near the cell membrane. One hundred and twenty min after pulse, SEG was seen in RAB-7 negative vesicles. (**C**) Only 20 min after the 1 h pulse, SEG could be detected in the lysosomal compartment surrounded by LAMP-2). At 60 min, co-localization with SEG was also apparent. However, at 120 min, there was only trace co-localization of LAMP-2 and SEG, but clusters of SAg could be seen on the cell membrane, which remained up to 240 min of culture. Figures show a representative experiment of 4–5.

Laser scanning confocal microscopy was also used to investigate the location of SEG after DC uptake. For this purpose, we used markers of early and late endosomes and lysosomes. DCs were cultured in poly-L-Lys treated slides, pulsed for 1 h at 37°C with SEG, washed, and cultured from 0–240 min. After washing and fixing, cell immunolabeling was performed in the presence of saponin for intracellular staining with rabbit polyclonal antibodies against the early endocytic specific marker, EEA-1; the late endosomal marker, Ras-related protein RAB-7; or the lysosomal-associated membrane protein-2, LAMP-2; and mouse anti-SEG, at 0, 20, 40, 60, 90, 120, 180 and 240 min end points. Normal rabbit and mouse polyclonal antibodies were used as negative control. Cells were then incubated with FITC-labeled anti-rabbit and rhodamine A-labeled anti-mouse antibodies for analysis by confocal microscopy. **Fig. 3A** shows that, immediately after the pulse (0 min), SEG could be observed intracellularly in the endomembrane compartment, co-localizing with EEA-1. Twenty min later, SEG still co-localized with EEA1, but at 40 min after the pulse, little or no EEA1 co-localized with SEG, although the SAg remained in vesicles. Incubation times beyond 40 min after the pulse showed no co-localization of EEA1 and SEG (data not shown). Co-localization of SAg with the late endosomal-specific RAB-7 GTPase started at 20 min when large and strongly stained vesicles could be seen (**Fig. 3B**). Sixty min after the pulse, SEG co-localized in vesicles with RAB-7 near the cell membrane; these vesicles were not seen at 120 min, although traces of SEG were still visible in intracellular compartments. When experiments were conducted analyzing SEG co-localization with LAMP-2, co-localization also began at 20 min after the 1 h pulse (**Fig. 3C**), was intense at 60 min, and decayed at 90 min (not shown). At 120 min after the pulse, there was minimal co-localization of LAMP-2 and SAg, which was absent at 240 min; however, in both cases, the SAg could be found in clusters near the cell membrane.

Shorter pulses with SEG were tested, including pulses of 20 and 40 min instead of 60 min, and co-localization with the different markers was studied at similar time points as previously. We observed that shorter pulses only resulted in less SAg incorporated, but did not affect co-localization patterns, times of progression, or the length of time SEG remained inside the different kind of vesicles (not shown).

### SAg Co-localizes with MHC-II Molecules on the DC Cell Surface and in Vesicles

**Figure 4:**
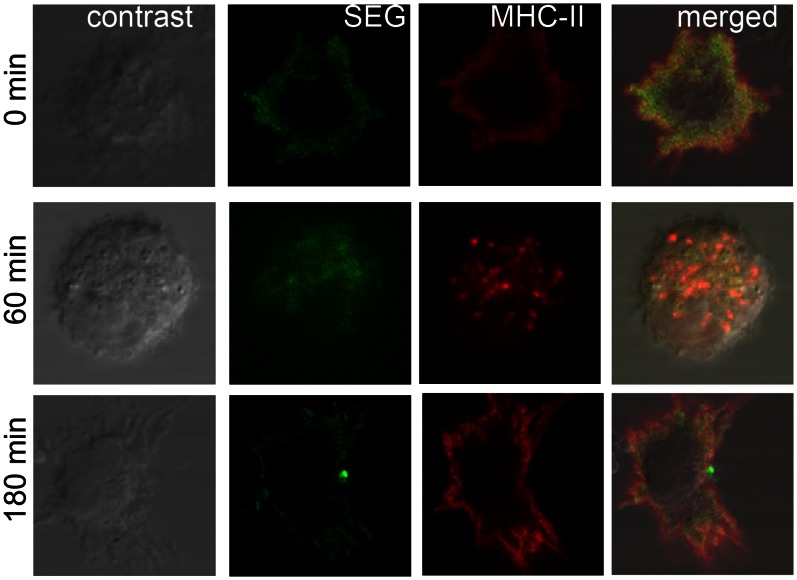
SEG co-localizes with MHC-II at the cell membrane and in vesicles. DCs were cultured in poly-L-lysine treated slides, pulsed 1 h at 37°C with SEG-FITC, washed four times and cultured for different periods (0–240 min). Cells were then fixed with paraformaldehyde and permeabilized with saponin for immunolabeling with a phycoerythrin-Cy5-labeled monoclonal antibody against mouse I-A and I-E MHC-II. A control of non-pulsed cells was performed in parallel. Confocal images were captured using a PlanApo 60× Oil AN1.40 lens. After 1 h pulse, SEG showed a dispersed pattern, with some clusters, near the cell membrane. Strong staining of MHC-II was observed at a similar location. At 60 min, SEG co-localized with MHC-II in vesicles. One hundred and eighty min later, clusters of MHC-II and SEG were observed, in addition to a scattered pattern for both SEG and MHC-II close to the cell membrane. Figure shows a representative experiment of 5.

Since the current paradigm holds that SAgs simultaneously bind TCR on the T cells and MHC-II on the APCs, we next analyzed whether SAg uptake was related to the presence of MHC-II on DCs. We used a monoclonal antibody which recognizes both I-A and I-E mouse MHC-II molecules. **Fig. 4** shows that after a 1 h pulse (time 0) SEG and MHC-II co-localized in the cytoplasm close to the cell membrane. Sixty min later, both molecules co-localized inside vesicles in the perinuclear area, and after 180 min, both SEG and MHC-II were located in the periphery of the cell membrane in a scattered pattern, despite also colocalize in vesicles at that time. Since co-localization does not necessarily means that both molecules traffic bound, we attempted to precipitate SEG bound to MHC-II using polyclonal antibodies against either SEG or MHC-II without success at any time (0–240 min) after DCs were exposed for 1 h to the SAg. We also attempted to cross-link both molecules at different times with the cross-linker bis(sulfosuccinimidyl)suberate (BS3), but without success (not shown).

### DCs are Able to Release Incorporated SAg

**Figure 5:**
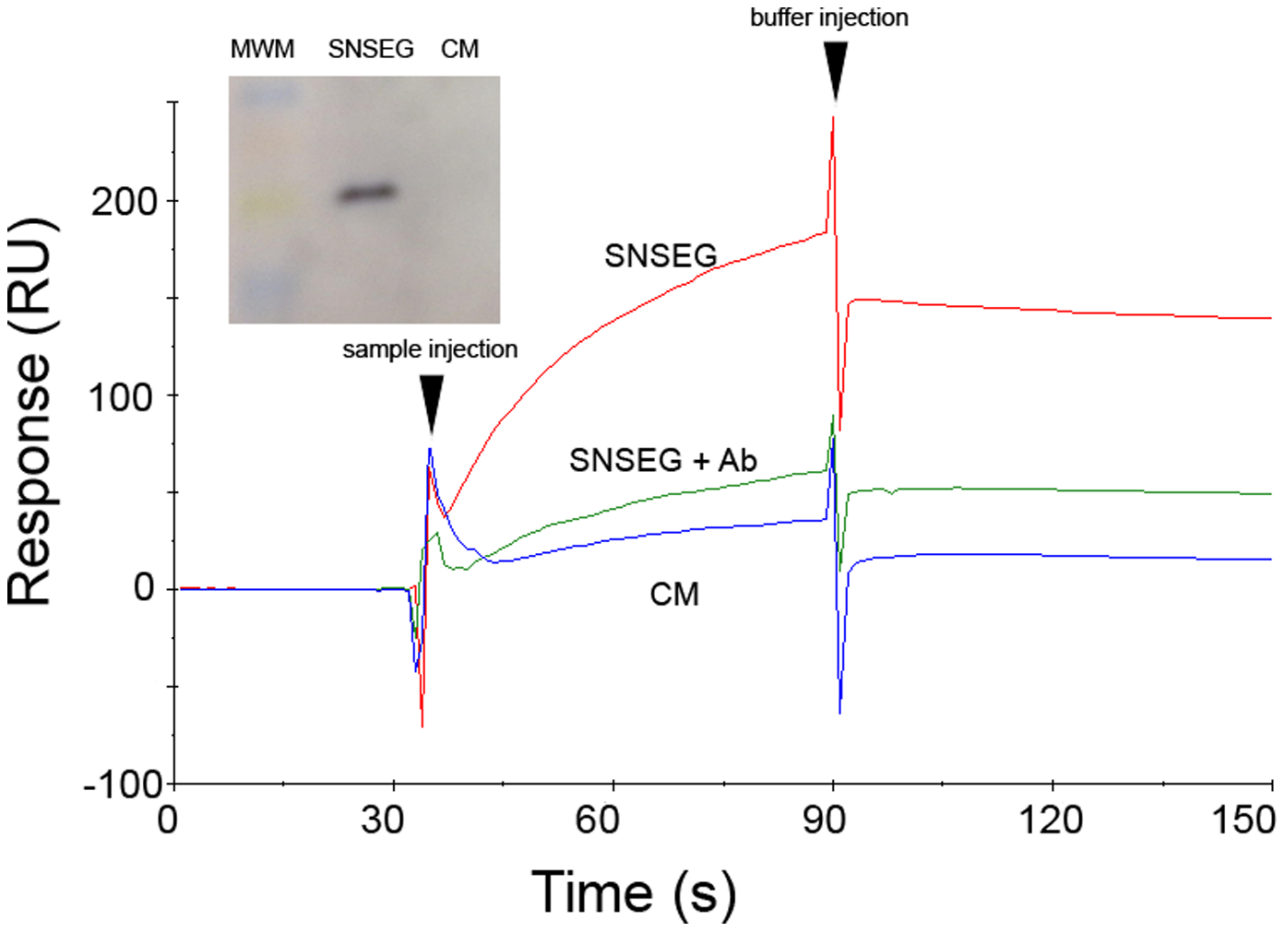
Analysis of SAg uptake by DCs and release into the medium by SPR. DCs were incubated in the presence, or absence, of 100 ug/mL SEG for 1 h, washed four times, and cultured in fresh medium for 3 h (SNSEG). Supernatant SNSEG diluted two-fold in fresh medium, or DC culture medium (CM) were injected over immobilized TCR Vβ8.2 and responses recorded as a function of time for 60 sec (30–90 sec), and then buffer was injected for dissociation. In addition, SNSEG diluted two-fold in fresh medium were neutralized with mouse anti-SEG (SNSEG+Ab) and injected over immobilized TCR Vβ8.2. Inset: Immunoblot of supernatant from DCs incubated in the presence (SNSEG) or absence (CM) of SEG. MWM: molecular weight markers. Figure shows a representative experiment of 3.

Since co-localization experiments showed that, after uptake and inclusion in different intracellular vesicles, SAgs were directed to the cell membrane as early as 1 h after pulse (**Fig. 3**), we next analyzed whether DCs were able to release the SAg. DCs were loaded with SEG for 1 h, carefully washed four times, and incubated with fresh medium for 3 h. Supernatants were collected and analyzed by SPR on a Biacore T100 biosensor. The SEG-specific ligand TCR Vβ8.2 was immobilized on a CM5 chip surface. A surface with no protein immobilized was used as control. Supernatants of cell pulsed with SEG, or not, were injected over these surfaces for 60 sec. **Fig. 5** shows strong binding of SEG to its specific Vβ8.2 ligand. After 3 h incubation, although the SAg was released to the medium, DCs still contained SEG in their vesicles (not shown). In order to further demonstrate that the SPR results were due to specific binding of SEG, we neutralized the supernatant with rabbit anti-SEG and passed the supernatant over the CM-5 chip surfaces with or without Vβ8.2. **Fig. 5** shows that the neutralized supernatant was unable to bind immobilized Vβ8.2. The inset shows an immunoblot in which SEG in the supernatant was identified by specific rabbit antibodies.

### DCs Pulsed with SEG Trigger Superantigen-Specific Response in Splenocytes

**Figure 6:**
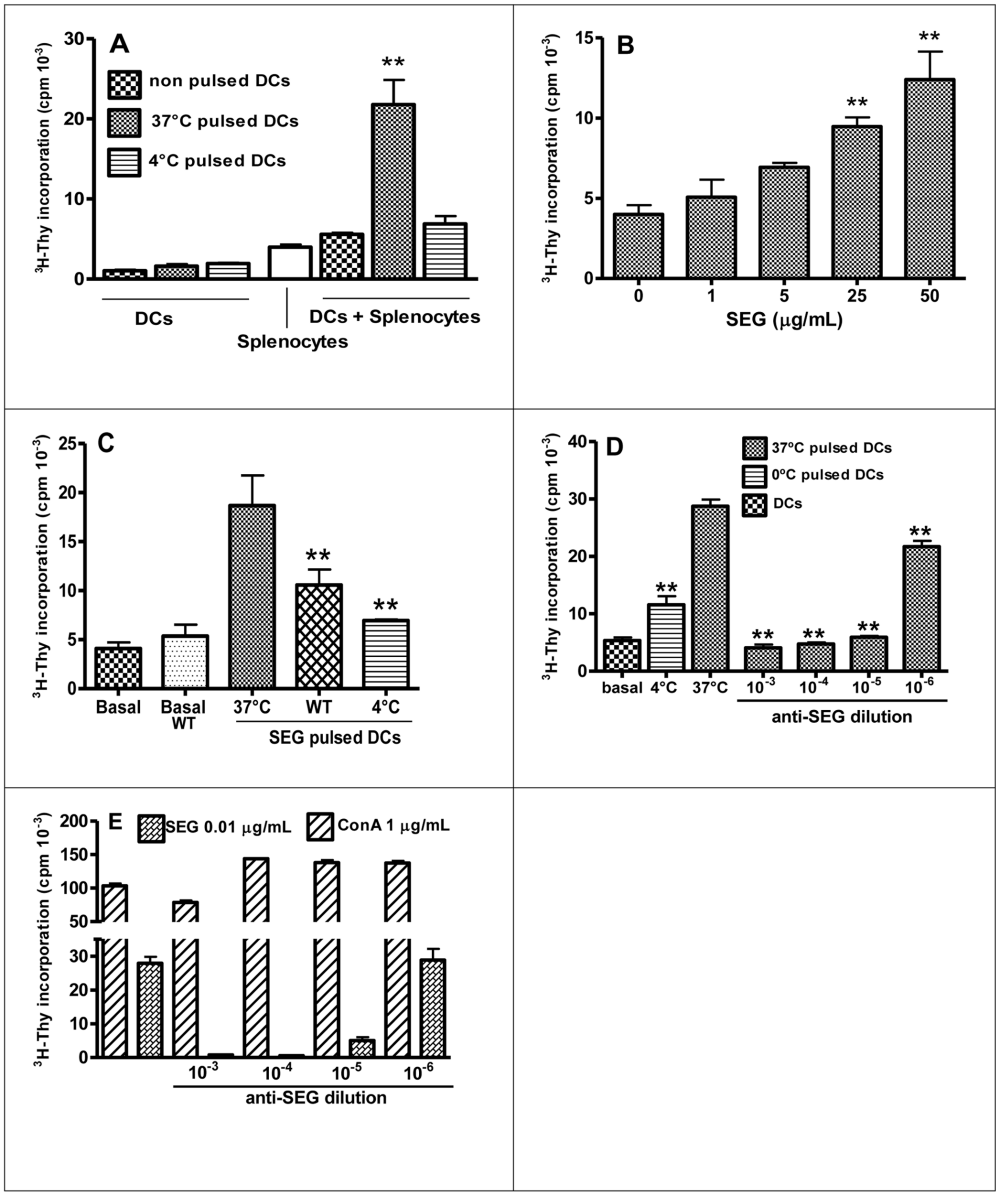
DCs induce superantigenic response after SEG uptake. DCs were pulsed 1 h at 37°C or 4°C with SEG, washed, and co-cultured with isogenic splenocytes in triplicate; controls of DCs with no addition of splenocytes were conducted in parallel. (**A**) ^3^H-Thy incorporation for DCs, splenocytes, and DCs plus splenocytes. (**B**) DCs were pulsed with different concentrations of SEG for a dose response curve, and co-cultured with splenocytes in triplicates. (**C**) Splenocyte proliferation induced by SEG-pulsed DCs and SEG-pulsed DCs in the presence of Wortmannin, performed in triplicates. (**D**) Rabbit anti-SEG serum was added to the cultures of pulsed DCs and splenocytes to specifically block SEG function. (**E**) Splenocytes were cultured in the presence of 0.01 µg/ml SEG or 1 µg/ml ConA, with the addition, or not, of different dilutions of rabbit anti-SEG serum. One representative experiment is shown of 3 to 5 experiments. **p<0.01.

To determine whether SAgs exposed on the DC cell membrane are able to induce a superantigenic response, mixed culture with splenocytes were carried out. DCs were pulsed with SEG as previously described, and co-cultured for an additional period of 72 h with syngeneic splenocytes. ^3^H-Thy incorporation for DCs, splenocytes, or DCs plus splenocytes showed significant differences between proliferation induced by 37°C pulsed DCs *vs*. basal non-pulsed DCs, while 4°C pulsed DCs showed no increment in proliferation (**Fig. 6A**). ^3^H-Thy incorporation assays revealed that DCs pulsed with different concentration of SEG efficiently induced cell proliferation in a dose-dependent manner (**Fig. 6B**). Splenocyte proliferation induced by pulsed DCs was significantly reduced when SEG was added in the presence of WT (72±14% inhibition) or at 4°C (82±8% inhibition) (**Fig. 6C**), which demonstrates the requirement of SAg incorporation for eliciting a proliferative response. To demonstrate that splenocyte proliferation was due to a superantigenic effect, we carried out experiments with the addition of an SEG-specific rabbit polyclonal antiserum to the co-cultures. The results showed that rabbit anti-SEG abrogated splenocyte proliferation induced by pulsed DCs, even at high antibody dilutions (1×10^−5^) (**Fig. 6D**). Additionally, to rule out any hypothetical impairment of cell function caused by the antiserum, splenocytes were cultured with SEG (0.01 µg/ml) or ConA (1 µg/ml), with or without anti-SEG. Antibodies specifically blocked superantigenic activity, whereas proliferation induced by the mitogen Con A was not blocked at any antiserum dilution (**Fig. 6E**).

### SEG-Pulsed DCs Specifically Stimulate Lymphocytes Bearing Vβ8.2 *in vivo* and *in vitro*

**Figure 7:**
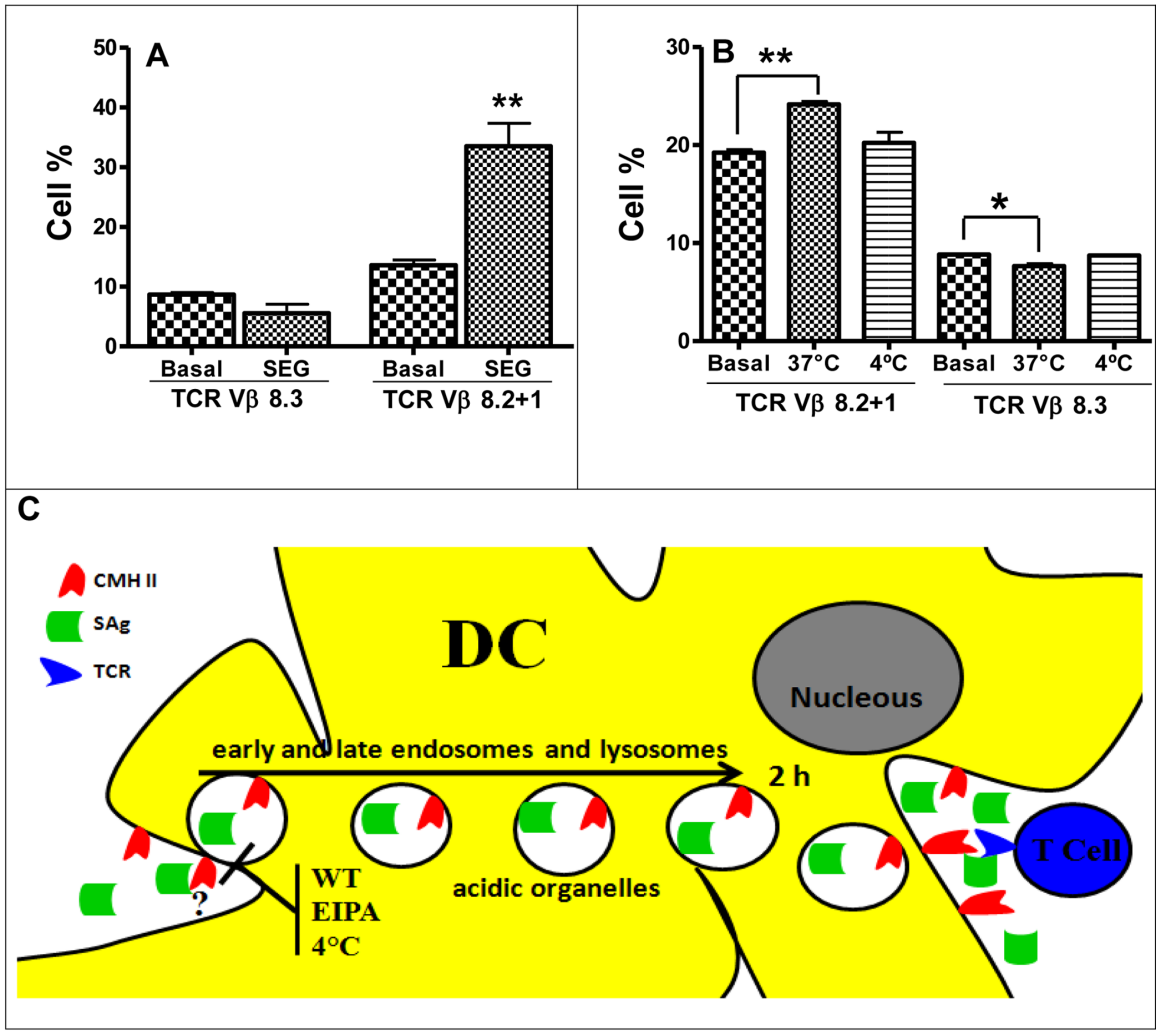
After SEG uptake, DCs stimulate lymphocytes bearing Vβ8.2 TCR. (**A**) DCs were pulsed 1 h at 37°C with SEG, washed, and co-cultured with isogenic splenocytes. FACS analysis of T cell populations was conducted with PE-conjugated anti-Vβ8.2+1 TCR and PE-conjugated anti-Vβ8.3 TCR monoclonal antibodies (BD Pharmingen). A control of non-pulsed DCs and splenocytes was conducted in parallel. (**B**) DCs pulsed at 37°C or 4°C with 50 µg/ml SEG were injected intradermically into BALB/c mice foot pads. Control animals were injected with non-pulsed DCs in parallel. FACs analysis with anti-Vβ8.2+1 and anti-Vβ8.3 was conducted on cells recovered from popliteal lymph nodes after 48 h. One representative experiment is shown of 3. *p<0.05 and ** p<0.01. (**C**) **Schematic diagram depicting SAgs uptake and trafficking in DCs.** After incorporation by macropinocytosis, SAgs can be located in early and late endosomes and lysosomes, as biologically active molecules, and then are recycled to the cell membrane. After trafficking, SAgs are capable of stimulate T cells bearing the proper TCR, triggering lymphocyte proliferation.

To further confirm that the observed proliferation corresponded to the superantigenic effect of SEG, we analyzed populations of lymphocytes bearing different Vβ chains in mixed cultures. SEG has been reported to stimulate the proliferation of mouse T cells expressing Vβ8.2, Vβ7, and Vβ9 [10], [22]. We selected Vβ8.2 and Vβ8.3 TCRs as representative of populations stimulated and not stimulated by SEG, respectively. Mixed cultures were performed as previously described. After 48 h of culture, cells were immunolabeled with PE-conjugated anti-Vβ8.2 or anti-Vβ8.3 antibodies. FACS analysis revealed a significant increase in the percentage of Vβ8.2+1 bearing T cells *vs.* basal level, while Vβ8.3^+^ T cells were similar to control (**Fig. 7A**). A reduction of the TCR Vβ8.3 cell population compared with basal levels is often observed in superantigenic responses, and is probably due to an increase in the percentages of other T cell populations.

We next analyzed whether stimulation of T cells bearing Vβ8.1+2 by SAg pulsed-DC could be reproduced *in vivo*. DCs pulsed with SEG at 37°C and 4°C were intradermically injected into the mice foot pad of BALB/c mice. After 48 h, mice were sacrificed and their popliteal lymph nodes removed for analysis of Vβ8.2+1 and Vβ8.3 T cell populations. FACS analysis showed a significant increase of Vβ8.2^+^ T cells in lymph nodes stimulated with DCs pulsed at 37°C, while DCs pulsed at 4°C were unable to stimulate Vβ8.2+1^+^ T cells (**Fig. 7B**).

## Discussion

Immature DCs use both macropinocytosis and receptor-mediated endocytosis to internalize soluble antigens efficiently. Usually, antigens are rapidly broken down following endocytosis. However, DCs also have the capacity to store unprocessed conventional antigens for long periods and to release them to the cell surface or to the extracellular medium [34]–[37]. Our present results indicate that DCs effectively take up SAgs, retain unmodified SAgs in endosomes, and finally release them to the extracellular medium. **Fig. 7C** shows a schematic diagram depicting SAgs uptake and trafficking in DCs. We hypothesize that this trafficking mechanism might promote the efficient encounter of SAgs with both DCs and T cells in lymph nodes.

The current paradigm for T cell activation by SAgs proposes direct and simultaneous interaction of SAg with TCR on the surface of the T cell, and with MHC-II on the surface of the APC [1], [38]. The activation of both APC and T cell produces a cytokine storm that can lead to TSS. However, little is known about the sites where SAgs encounter their specific ligands and how they reach those sites. DCs are specialized APCs present in all tissues which, after activation by antigens, have the ability to migrate to the next lymph node where they encounter circulating T cells that are looking for specific antigens to became effector T cells. The idea of SAgs interacting with T cells in peripheral tissues or in the blood, where they can only encounter a very limit number of T cells, is unlikely, whereas the idea of SAgs reaching lymph nodes through which every T cell will pass is more consistent with the known ability of SAg to elicit powerful T cell activation. Thus, lymph nodes would be the expected site for SAg to interact with their cognate ligands and activate T cells bearing particular Vβ regions. In a natural infection with SAg-producing bacteria, the draining lymph node of the infection site would be the most likely encounter site; SAgs could reach it as soluble antigen in lymphatic fluid. Here, we tested an alternative route for SAgs to encounter their specific ligands.

Immature DCs have an extraordinary ability to sample the surrounding environment by endocytosis. They use two main mechanisms for antigen capture: macropinocytosis, which occurs in a constitutive fashion in DCs and allows continuous internalization of antigens present in the fluid phase, and receptor-mediated endocytosis, which involves the internalization of antigens and pathogens after clustering of specific receptors [39], [40]. We initially analyzed *in vitro* SAg uptake by DCs and found that *S. aureus* SEG and SEI, as well as *S. pyogenes* SSA, were efficiently incorporated in a dose-dependent manner. This process was inhibited by WT and EIPA, an ion exchange inhibitor that inhibits membrane ruffling, strongly suggesting that uptake is by macropinocytosis [41]. Uptake of SEG, SEI or SSA does not induce the maturation of DCs, which has also been recently demonstrated for SEB and SEA [33]. Conversely, Muraille et al. [30] found that intravenous injection of SEB or TSST-1 in mice induces maturation of DCs in the spleen, but that this was a consequence of *in vivo* T cell stimulation produced by the simultaneous binding of the exotoxins to TCR and MHC-II. Similarly, *in vivo* administration of SEA- increased the number of plasmocytoid DCs in secondary lymphoid organs and induced CD86 and CD40 expression, although that effect strictly required the presence of IFN-γ [31]. However, incubation of immature DCs with SAg alone neither changed cellular morphology nor did DCs became mobile [28]. Thus, we speculate that during the course of infection by *S. aureus* or *S. pyogenes*, the maturation of DCs and their migration to the lymph nodes would be induced by components other than the SAgs themselves, including bacterially-derived pathogen-associated molecular pattern (PAMPs) such as lipoteichoic acid and peptidoglycans [42], [43].

We next analyzed the fate of SAgs after DC uptake. We used laser scanning confocal microscopy to examine the subcellular localization of SAgs after a 1 h pulse. SEG co-localized preferentially with the LysoTracker probe in a few large acidic organelles as soon as 60 min after the pulse, and later in multiple small endosomal vesicles. EEA1 is a membrane-bound protein component specific to the early endosome and is essential for fusing early endocytic vesicles and for phagosomal maturation [44]. SEG was found in vesicles identified by the EEA1 protein from time points 0 to 40 min after the 1 h pulse. By contrast, a mutant of the streptococcal superantigen SMEZ-2 conjugated with OVA seemed to follow a different intracellular pathway, since it remained in a EEA1-negative compartment at 10 and 30 min after pulsing [26]. This effect cannot be attributed to conjugation because OVA alone co-localized with EEA1 at both time points. Further investigation is needed to determine whether segregation to a different intracellular compartment is a unique property of the SMEZ-2-OVA conjugate, or a particularity of some SAgs.

RAB-7 is a key regulator in endo-lysosomal trafficking which governs early-to-late endosomal maturation, microtubule directed endosomal migration and positioning, and endosome-lysosome transport [45]–[48]. Co-localization of SEG with RAB-7 began 20 min after a 1 h pulse and persisted for at least 40 more min. Similarly, SEG co-localized with LAMP-2, a marker protein for lysosomes, from 20 min after a 1 h pulse until at least 100 min. Strikingly, 60 min after a 1 h pulse, SEG co-localized with RAG-7 or LAMP-2 near the DC cell membrane (**Fig. 3C and D**). LAMP-2 has been described as mainly associated with lysosomes, but can also shuttle between early endocytic compartments and the plasma membrane [49], which may explain co-localization in places where the SAg will be during trafficking in DCs.

We also analyzed whether SAgs maintain superantigenicity after DC incorporation. We found that SEG in the acidic environment of the endosomal/lysosomal compartment (**Fig. 2** and **Fig. 3**), SEG in the membrane 2 h later, as well as SEG released into the medium after 3 h (**Fig. 5**), was biologically active. Moreover, the SAg retained enough structure to allow recognition by polyclonal mouse and rabbit antisera during the entire process of uptake, inclusion in diverse vesicles, and exposure on the membrane, in addition to being able to stimulate specific T cells bearing particular TCRs. These results strongly suggest that SAgs traffic through DCs in intact form. This is not surprising, since SAgs are known to be very stable and highly resistance to proteases, and can resist temperatures of 60°C or higher, as well as pH extremes of 2.5 to 11 [50], [51].

Since SAgs bind MHC-II, we analyzed whether SAg uptake by DCs was related to the presence of MHC-II on the cell membrane. Even though we used immature DCs which do not have high expression of MHC-II, SEG co-localized with MHC-II near the cell membrane 1 h after pulsing (**Fig. 4**). Sixty min later, both molecules were inside vesicles and 180 min after the pulse, both molecules could be seen near the cell membrane, which is in agreement with the intracellular trafficking of SEG reported here. However, attempts to precipitate SEG bound to MHC-II were unsuccessful at any time after DCs were exposed to the SAg, using polyclonal antibodies against either SEG or MHC-II. We also attempted to cross-link both molecules at different times (0–240 min after a 1 h pulse of SAg) with the cross-linker bis(sulfosuccinimidyl)suberate (BS3) that we used previously to determine the stoichiometry of the Ly49A-H-2D^d^ complex [52], but without success (not shown). The failure to demonstrate formation of a SEG-MHC-II complex in murine DCs could be due to lack of contact with MHC-II during entrance and trafficking of the SAg, or to a fast dissociation rates (short half life) of the SEG-MHC-II interaction. Although the affinity and kinetics of SEG binding to murine MHC-II have not been measured, we previously determined by analytical ultracentrifugation that SEG binds the human MHC-II molecule HLA-DR1 with relatively low affinity (*K_D_ = *32 µM) [12]. In addition, all SAgs examined to date display higher affinity for human than murine MHC-II molecules [53].

We next analyzed whether the incorporated SAg, was able to stimulate T cell proliferation. *In vitro* experiments showed that SEG-pulsed DCs could stimulate splenocytes from syngeneic mice expressing Vβ8.2 TCRs. *In vivo* experiment gave similar results when SEG-pulsed DCs were introduced into the foot pad and the draining lymph node was analyzed for Vβ8.2 T cell proliferation. Manipulation of DCs after the SAg pulse or generation of endogenous danger-associated molecular patterns (DAMPS) caused by needle injury may explain the migration to the lymph node in the absence of a maturation stimulus. In addition to MHC-II and TCR, recent data strongly suggest that other bacterial and host components play an important role in mediating SAg effects. Thus, peptidoglycan-embedded molecules interacting with Toll-like receptor 2 have been shown to induce interleukin-10 production and apoptosis of APCs, thus downregulating T cell activation and preventing SAg-induced TSS [54]. In addition, it has been demonstrated that the principal co-stimulatory receptor CD28, constitutively present on T cells, is a third SAgs receptor that is essential for induction of TSS [55], [56].

Recently, APCs have been shown to use distinct endocytosis mechanisms to simultaneously introduce soluble antigen into separate intracellular compartments for degradation into peptides and loading into MHC-I or MHC-II molecules for presentation to CD8^+^ or CD4^+^ T cells, respectively [57]. Here we have shown that SAgs escape and are released from DCs unharmed. How SAgs are diverted from that fate and whether it is a property of the SAg or a particular uptake mechanism of DCs remains to be clarified. In this regard, Le Roux et al. [58] found that immature DCs are able to store unprocessed antigens and release them in the lymph node to be taken up by B cells. Based on our results, the uptake of SAgs and their secretion in a form able to stimulate specific T cells may represent a particular case of a more general mechanism for transporting and presenting antigens in native form to T and B cells in lymph nodes.

## Materials and Methods

### Ethics Statement

All procedures requiring animals were performed in agreement with institutional guidelines and were approved by Review Board of Ethics of IDEHU, CONICET, and conducted in accordance with the guidelines established by the National Research Council of Argentina.

### Superantigens

Streptococcal SSA and staphylococcal SEI and SEG were produced and purified as previously described [59], [10]. Briefly, proteins cloned in pET-26b were expressed in *Escherichia coli* BL21 cells and purified by Ni^2+^-NTA column chromatography, followed by S-200 molecular exclusion. Proteins were treated with agarose-polymixin B (Sigma Aldrich, St. Louis, MO) to remove LPS traces and absence of LPS was assessed by Limulus test. The SAgs SEG, SEI and SSA were labeled with FITC for endocytosis assays in conjugation buffer (50 mM Na_2_CO_3_/NaHCO_3_ pH 9, 150 mM NaCl) and free dye was removed using a G25 molecular exclusion column. The F/P ratio was estimated by measuring Abs_490_ and Abs_260_. F/P ranged between 2.6 and 3.2, and biological activity was corroborated by spleen cell proliferation measured by ^3^H-Thy incorporation at 72 h.

### Polyclonal Antibodies to SEG

Polyclonal serum anti-SEG was obtained by immunizing mice and rabbits with purified protein and Freund´s adjuvant as previously described [10].

### Mice and Culture Conditions

Experiments were conducted using 8–12 week old male BALB/c mice. They were housed 4–6 per cage and kept under standard conditions. Unless otherwise specified, cell cultures and incubations were maintained at 37°C in a humidified atmosphere with a 5% CO_2_ atmosphere, using complete medium composed of RPMI 1640 supplemented with 10% heat-inactivated fetal bovine serum (Gibco-BRL Life Technologies, Grand Island, NY), 2 mM L-glutamine, 1 mM pyruvate, 100 U/ml penicillin, and 100 µg/ml streptomycin.

### Dendritic Cells

DCs were obtained from the bone marrow of BALB/c mice as previously described [60]–[62], with minor modifications. Briefly, femora and tibiae from mice were removed and stripped of muscles and tendons; both ends were cut and bone marrow was flushed with RPMI 1640 medium using a 21-gauge needle and syringe. Cell clusters were dissociated by repeated pipetting and 0.45 M ammonium chloride was added for red cell lysis. After washing the cell suspension once in RPMI 1640, cells were resuspended in complete medium and cultured at a concentration of 1×10^6^ cells/ml for 7 days in complete medium supplemented with 20% conditioned medium from a GM-CSF producing cell line (J558L myeloma cell line transfected with GM-CSF cDNA) [63]. Cultures were fed at day 3 and 5 as follows: after gently removing 75% of the medium, cells were centrifuged 10 min at 600 xg, the pellet was resuspended in fresh medium with GM-CSF, and the cell suspension was returned to the dish. At day 7, cells were evaluated for expression of CD11c and MHC-II by flow cytometry, yielding 70–83% of cells positive for both markers. Cell viability was also estimated by trypan blue exclusion to be >95% in all experiments.

### SAg-FITC Endocytosis

DCs were incubated at 1×10^6^ cells/0.2 ml in complete medium, and SAgs-FITC were immediately added to final concentrations of 50, 25, 10, 5 or 1 µg/ml SEG-FITC for 1 h at 37°C and at 4°C under 5% CO_2_. Then, cells were washed 4 times in cold PBS, and analyzed by FACS, with the addition of trypan blue at a final concentration of 200 µg/ml for quenching of external fluorescence [64], [65]. This concentration was determined as the most efficient in previous experiments in which DCs were stained with FITC-mAb directed to cell surface CD11c, as described [66]. In order to determine the mechanism involved in SEG uptake, and in a separate set of experiments, the macropinocytosis inhibitors wortmannin (WT) and 5-(N-ethyl-N-isopropyl) amiloride (EIPA) were added at final concentrations of 100 nM and 50 µM, respectively, 15 min before SAg-FITC at 50 µg/ml. A control with DMSO was performed, and cells were analyzed by FACS as previously described.

### Fenotipic Assay and OVA-FITC Endocytosis

DCs (1×10^6^ cells/ml) were incubated with 100 µg/ml of SSA, SEG and SEI or 1 µg/ml of E. coli LPS (Sigma Aldrich, St. Louis, MO) for 24 h at 37°C in complete medium. MHC-II, CD80 and CD86 were measured by FACS and compared to basal expression in DCs incubated without SAg. Isotype controls were performed. For the OVA-FITC endocytosis assay, DCs were incubated with SAgs or LPS as previously indicated, washed and incubated with 100 µg/ml of OVA-FITC (obtained as described above for SAgs-OVA) for 3 h at 37°C. A control with OVA-FITC at 4°C (background control) was performed.

### Flow Cytometry

We examined DC fenotype by direct immunofluorescence staining using FITC-labeled mAbs to I-Ad (MHC-II) or CD11c and phycoerythrin-labeled antibodies to CD11c, I-Ad, CD40, CD80 or CD86 (BD PharMingen, San Diego, CA). Stained cells were analyzed by flow cytometry in a PASS III flow cytometer (Partec, Görlitz, Germany). Twenty thousand events were acquired for each sample and data analysis was performed using the WinMDI software program. Results were expressed as percentage of positive cells or MFI as appropriate.

### Presence of SEG in the Endosomal/Lysosomal Fraction and on Cell Membrane

To evaluate the presence of SAgs in the intracellular compartment, DCs were incubated for 1 h with SEG at 37°C or 4°C as control, washed, and cultured for additional periods from 0 to 24 h. At each endpoint, cells were washed and disrupted in order to isolate the endosomal/lysosomal compartment as previously described [67] with slight modifications. Briefly, 10^6^ cells were lysed with hypotonic solution 20 mM Hepes, 0.5 mM EDTA, 0.25 M sucrose and 0.1% BSA, plus mechanical disaggregation by 20 successive passing through a 27 G1/2 syringe. Lysates were then centrifuged 6 min at 150×g, and supernatant recovered for an additional spin at 13000×g for 10 min. Pellet containing endosome/lisosome fraction was kept at −20°C until proliferation assays were performed. Then, the endosome/lysosome fraction was disrupted by repeated cycles of freezing and thawing, resuspended in 0.3 ml of complete medium, and exposed to syngeneic splenocytes in triplicate to assess cell proliferation activity by ^3^H-Thy incorporation as a biological method to detect SAgs. The proliferation index (PI) was calculated as cpm/basal cpm. To analyze the presence of SAg on the cell membrane, indirect immunolabeling was carried out. SEG-pulsed DCs cultured as indicated before, were washed and cultured to each endpoint. Indirect immunolabeling was carried out using mouse polyclonal anti-SEG antibodies and anti mouse-FITC, as second antibody; and cells were analyzed by FACS.

### Co-localization Assays

Co-localization assays between SEG and different vesicle markers (EEA1, RAB-7 and LAMP-2), were performed as follows. DCs were attached to slides previously treated with poly-L-lysine (0.01%) and cultured with 50 µg/ml of SEG for 1 h. Then, cells were washed four times with RPMI 1640 to remove non-internalized SAg. Afterwards, complete medium was added to the slides and cells cultured further in order to allow them to evolve for different periods of time: 0, 20, 40, 60, 90, 120, 180 and 240 min. Controls of DCs without SEG were performed at every end point. When these different periods were concluded, cells were washed twice with PBS and fixed with 2% paraformaldehyde at 4°C. The cells were then washed with PBS, blocked with 0.01% glycine for 1 h, and permeabilized with 0.1% saponin for 10 min for immunofluorescent labeling. Incubation for 1 h at room temperature with the primary mouse polyclonal serum to SEG concurrently with polyclonal rabbit anti-EEA1, anti-Rab7, anti-Lamp2, (Sigma Aldrich, St. Louis, MO) was followed by 1 h incubation with secondary antibodies, FITC-conjugated anti-rabbit, and rhodamin-conjugated anti-mouse (Jackson ImmunoResearch). Fluorescently labeled cells were fixed with paraformaldehyde at 4°C, and cover slides mounted using anti-fade mounting media (Vector Laboratories). For co-localization of SEG with acidic organelles, DCs were cultured in poly-L-lysine treated slides, pulsed 1 h at 37°C with 50 µg/ml SEG-FITC or OVA-FITC and 50 µM Lysotracker RED (Molecular Probes, Invitrogen), washed and cultured further for different time periods (0–240 min). For SEG and MHC-II co-localization DCs were pulsed with SEG-FITC, washed, and cultured for different periods as described above. Cells were then fixed with paraformaldehyde and permeabilized with saponin for immunolabeling with phycoerythrin-Cy5-labeled mAb against mouse I-A and I-E MHC-II. A control with non-pulsed cells was performed in parallel. Image capture was performed using a Nikon C1 confocal laser scanning microscope with a PlanApo 60× Oil AN1.40 lens. For detection of rhodamine, a 543 nm laser and 490 nm laser for FITC were used. A 546-nm excitation wavelength and a 590-nm emission wavelength were used for LysoTracker.

### Mixed Cultures

In order to determine the capacity of DCs pulsed with SEG to induce superantigenic responses, DCs were co-cultured with murine splenocytes for assessing total proliferation and stimulation of SEG-specific Vβ8.2+1 TCR bearing cells. DCs were pulsed 1 h with 50, 25, 10, 5 or 1 µg/ml SEG at 37°C, washed four times, counted in a Neubauer chamber and cell viability evaluated by trypan blue exclusion. Cells were added to round bottom 96 well plates in 0.2 ml of complete medium, at a responder cell-to-pulsed DCs ratio of 10∶1. Controls with non-pulsed DCs and cells pulsed at 4°C were also performed. Cultures were conducted at 37°C under CO_2_ for 72 h, and 1 µCi ^3^H-Thy was added for the last 16 h of culture to assess total proliferation. Cells were collected on glass fiber paper and cpm determined by liquid scintillation counting. Assays were conducted in triplicate, being shown a representative of three experiments. Additional assays were conducted in the presence of polyclonal rabbit anti-SEG, with parallel controls using ConA in the presence of similar concentrations of antibodies, to ensure that the splenocyte proliferation capacity was not abrogated. Proliferation of T cell populations bearing specific Vβ chains was evaluated as follows. Splenocytes and DCs pulsed 1 h with 50 µg/ml SEG at 37°C were co-cultured as previously described, harvested at 48 h, and direct immunolabeling performed separately with PE-conjugated anti-Vβ8.2+1 TCR and PE-conjugated anti-Vβ8.3 TCR (BD Pharmingen). Cells were evaluated by FACS to determine the percentage of blasts bearing each TCR, and compared with control. An additional control of blasts obtained from splenocytes stimulated with 1 µg/ml of the mitogen concanavalin A (ConA) was performed.

### *In vivo* Assays

DCs pulsed with 50 µg/ml SEG were introduced by intradermic injection into the foot pad of BALB/c mice. The left hind limb foot pad was sterilized with alcohol and slowly injected with 25 µl of cell suspension containing 0.2×10^6^ cells. Control animals were injected with non-pulsed DCs in parallel. After 48 h, mice were sacrificed by cervical dislocation, and their popliteal lymph nodes removed. Afterwards, single cell suspensions were obtained from each node by mechanical dislodging. Cells were immunolabeled separately with PE-conjugated anti-Vβ8.2+1 TCR and PE-conjugated anti-Vβ8.3 TCR, and the percentage of each population evaluated by FACS.

### Analysis of SAg Taken Up by DCs and Released to the Medium

DCs were incubated in the presence, or absence, of 100 µg/mL SEG for 1 h, washed four times and cultured in complete medium for 3 h. After that, cells were centrifuged and supernatants (SNs) diluted two-fold in fresh medium. Diluted SNs were passed over an immobilized TCR Vβ8.2 on a CM5 sensor chip and data recorded as a function of time by SPR analysis using a Biacore T100 instrument [4], [12]. As a control, diluted SNs were treated with an anti-SEG antiserum prior to injection over immobilized TCR Vβ8.2. In addition, immunoblotting was performed with SNs from DCs incubated in the presence or absence of SEG and revealed with rabbit anti-SEG polyclonal antibodies as previously described [10].

### Statistical Analysis

One-way ANOVA tests were performed to determine the significance of differences between means, and p<0.05 was taken as indicating statistical significance. In ^3^H-Thy incorporation assays, the proliferation index was compared with 2, and statistical significance was evaluated using *p*<0.05 in one sample test.

## Supporting Information

File S1 **Contains: Figure S1. Supplementary figure to** **Figure 1** **A and B. SAg incorporation in DCs.** (A) DC CD11c-PE isotype control; (B) CD11c expression and SEG incorporation in DC cultured at 37°C without SEG-FITC, and (C) with 50 µg/ml of SEG-FITC. Percentage of DC incorporating 50 µg/ml SEG-FITC in the presence of (D) WT, (E) EIPA or (F) at 4°C. **Figure S2. Supplementary figures to** **Figure 1D, E and F** **. SAg incorporation in DCs and cell activation markers.** Bone marrow DCs were pulsed 1 h with SEG-FITC, washed, and immunolabeled with anti CD11c-FITC. (D) Cell surface MHC-II molecules on DCs treated with SSA, SEG, SEI or LPS for 24 h, compared to non-treated basal control, and expressed as percentage of positive cells. (E) CD80 on SAgs- or LPS-treated DCs compared to non-treated. (F) Endocytosis of OVA-FITC by SAg or LPS pre-treated DCs. (I) CD86 on SAgs- or LPS-treated DCs compared to non-treated. (J) CD40 on SAgs- or LPS-treated DCs compared to non-treated. Figures show a representative experiment of 3–5. *p<0.05, **p<0.01. **Figure S3. DCs re-expose SEG on plasma membrane.** DCs were pulsed with SEG for 1 h, cultured further for the indicated times (0–240 min), and incubated with anti-SEG polyclonal antibodies and specific FITC conjugated antibodies without fixation and permeabilization treatment. The presence of SEG at the DC plasma membrane after 3 h confirms that the SAg was re-exposed on the cell membrane.
(DOCX)
