# Supplementary material for: Uptake and Intracellular Trafficking of Superantigens in Dendritic Cells
Source: PLoS One. 2013 Jun 14;8(6):e66244. doi: 10.1371/journal.pone.0066244 (PMC3682983; doi:10.1371/journal.pone.0066244)
Supplement: File S1 — Contains: Figure S1. Supplementary figure to Figure 1 A and B. SAg incorporation in DCs. (A) DC CD11c-PE isotype control; (B) CD11c expression and SEG incorporation in DC cultured at 37°C without SEG-FITC, and (C) with 50 µg/ml of SEG-FITC. Percentage of DC incorporating 50 µg/ml SEG-FITC in the presence of (D) WT, (E) EIPA or (F) at 4°C. Figure S2. Supplementary figures to Figure 1D, E and F . SAg incorporation in DCs and cell activation markers. Bone marrow DCs were pulsed 1 h with SEG-FITC, washed, and immunolabeled with anti CD11c-FITC. (D) Cell surface MHC-II molecules on DCs treated with SSA, SEG, SEI or LPS for 24 h, compared to non-treated basal control, and expressed as percentage of positive cells. (E) CD80 on SAgs- or LPS-treated DCs compared to non-treated. (F) Endocytosis of OVA-FITC by SAg or LPS pre-treated DCs. (I) CD86 on SAgs- or LPS-treated DCs compared to non-treated. (J) CD40 on SAgs- or LPS-treated DCs compared to non-treated. Figures show a representative experiment of 3–5. *p<0.05, **p<0.01. Figure S3. DCs re-expose SEG on plasma membrane. DCs were pulsed with SEG for 1 h, cultured further for the indicated times (0–240 min), and incubated with anti-SEG polyclonal antibodies and specific FITC conjugated antibodies without fixation and permeabilization treatment. The presence of SEG at the DC plasma membrane after 3 h confirms that the SAg was re-exposed on the cell membrane. (DOCX) [file pone.0066244.s001.docx]

**Supplementary Figures**

**Figure S1.** Supplementary figure to Figure 1 A and B.

|  |  |
| --- | --- |
|  |  |
|  |  |
| **Sag incorporation in DCs.** (**A**) DC CD11c-PE isotype control; (**B**) CD11c expression and SEG incorporation in DC cultured at 37°C without SEG-FITC, and (**C**) with 50 µg/ml of SEG-FITC. Percentage of DC incorporating 50 µg/ml SEG-FITC in the presence of (**D**) WT, (**E**) EIPA or (**F**) at 4°C. | |

**Figure S2.** Supplementary figures to Figure 1 D, E and F.

| **** |  |
| --- | --- |
|  |  |
| **** |  |
| **SAg incorporation in DCs and cell activation markers.** Bone marrow DCs were pulsed 1 h with SEG-FITC, washed, and immunolabeled with anti CD11c-FITC. **(D)** Cell surface MHC-II molecules on DCs treated with SSA, SEG, SEI or LPS for 24 h, compared to non-treated basal control, and expressed as percentage of positive cells. **(E)** CD80 on SAgs- or LPS-treated DCs compared to non-treated. **(F)** Endocytosis of OVA-FITC by SAg or LPS pre-treated DCs. **(I)** CD86 on SAgs- or LPS-treated DCs compared to non-treated. **(J)** CD40 on SAgs- or LPS-treated DCs compared to non-treated. Figures show a representative experiment of 3-5. **p*< 0.05, ***p*< 0.01. | |

**Figure S3.**

| **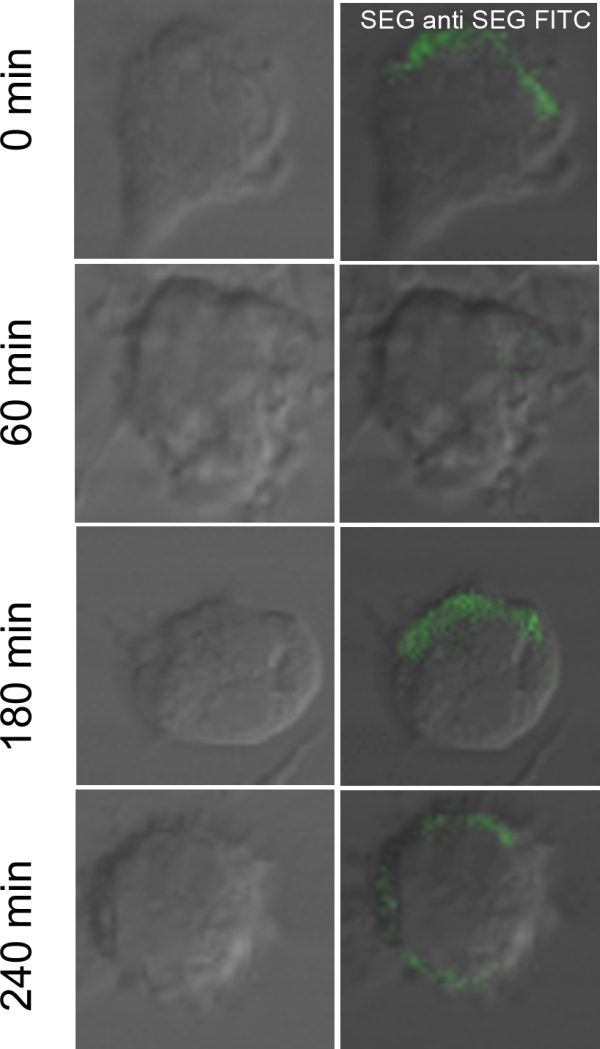** |
| --- |
| **DCs re-expose SEG on plasma membrane.** DCs were pulsed with SEG for 1 h, cultured further for the indicated times (0-240 min), and incubated with anti-SEG polyclonal antibodies and specific FITC conjugated antibodies without fixation and permeabilization treatment. The presence of SEG at the DC plasma membrane after 3 h confirms that the SAg was re-exposed on the cell membrane. |
